# A Study on Modified Bitumen with Metal Doped Nano-TiO_2_ Pillared Montmorillonite

**DOI:** 10.3390/ma12121910

**Published:** 2019-06-13

**Authors:** Jiao Jin, Bozhen Chen, Lang Liu, Ruohua Liu, Guoping Qian, Hui Wei, Jianlong Zheng

**Affiliations:** 1 School of Traffic and Transportation Engineering, Changsha University of Science and Technology, Changsha 410114, China; chenbozhen@stu.csust.edu.cn (B.C.); 2238544077@stu.csust.edu.cn (L.L.); wh@csust.edu.cn (H.W.); zjl@csust.edu.cn (J.Z.); 2 School of Minerals Processing and Bioengineering, Central South University, Changsha 410083, China

**Keywords:** bitumen, montmorillonite, metals doped, automobile exhaust, photocatalytic

## Abstract

TiO_2_ pillared montmorillonite (T/M) modifiers have been studied to alleviate the aging of asphalt pavement and degrade automobile exhaust, but the photocatalytic activity of ordinary TiO_2_ is not good enough. In this study, in order to improve the photocatalytic performance of T/M, different metal (Ce, Cu, Fe) doped modifiers were prepared based on T/M. Metal doped TiO_2_ pillared montmorillonite was prepared by the sol-gel method. The modifier was characterized by X-ray diffraction (XRD) and an Ultraviolet visible (UV-Vis) spectrophotometer. The results show that TiO_2_ with different metal ions successfully entered into the layer of organic montmorillonite (OMMT) to form a pillared structure. Compared with the undoped TiO_2_ pillared montmorillonite (T/M), the optical absorption edge of the metal doped TiO_2_ pillared montmorillonite has an obvious red shift. In addition, the influences of the different content of modifiers on the properties of the original bitumen and catalytic capacities for automobile exhaust were also investigated. The results show that Ce doped TiO_2_ (Ce-T/M) pillared montmorillonite has the best improvement in high temperature performance and ultraviolet (UV) resistance of bitumen. In the experiment of automobile exhaust degradation, the degradation law of NO and HC showed Cu-T/M > Ce-T/M > Fe-T/M > T/M. These three kinds of metal ions can effectively improve the photocatalytic degradation efficiency of T/M.

## 1. Introduction

With the increasing number of cars, the impact of automobile exhaust on the environment is becoming more and more serious. In practical road applications, heavy traffic also creates higher requirements for asphalt pavement. Common bitumen easily causes various road diseases because of its poor rheological performance, high temperature sensitivity, inadequate aging resistance, and other problems [1,2,3,4]. Therefore, it is of great significance for modern road construction to develop a kind of bitumen modifier that can not only improve the performance of asphalt pavement but also protect the environment and reduce automobile exhaust pollution [5,6].

Montmorillonite (MMT) is one of the most extensively studied mineral modifiers, which can enhance the high-temperature properties and aging resistance of bitumen. However, there are many inorganic ions in the montmorillonite layers, which show hydrophilic properties and poor dispersion in organic polymer materials. Incorporation of organic component in montmorillonite can solve this problem [7,8]. Organic montmorillonite (OMMT) has larger lamellar spacing, which makes it easier for organics and metal ions to enter the interlayer. Through organic modification, the montmorillonite can also change from hydrophilic to lipophilic and has good organic compatibility and dispersion [9,10,11]. Moreover, its unique lamellar structure can more easily be propped up by pillared compounds. It can be used to modify the structure of bitumen by using its interlayer characteristics, and the pavement performance of bitumen can be further enhanced after mixing with bitumen.

A series of nano-photocatalytic materials, for example TiO_2_, Fe_3_O_4_ and ZnO, have been widely used to adsorb and catalyze the degradation of pollutants [12,13,14]. However, the direct addition of nano-materials to bitumen is often ineffective, which is related to the compatibility and dispersion of materials and whether if it will form agglomeration. Therefore, the montmorillonite can be combined with the nano-materials to allow the nanoparticles to enter into the montmorillonite layer to form a pillared modified montmorillonite. This method is conducive to enlarging the interlayer spacing of montmorillonite, forming an exfoliated structure in the organic polymer materials, increasing the surface area of the materials and improving the catalytic performance and interlayer cation exchangeability of the materials [15,16].

TiO_2_ is one of the most common photocatalytic materials, but the catalytic activity of TiO_2_ is not good enough in the visible light range. Therefore, researchers use ion-doping method to improve catalytic performance, such as TiO_2_/montmorillonite composites [17,18]. In this paper, TiO_2_ pillared montmorillonite (T/M) was doped with Ce, Fe, and Cu ions to improve the performance of T/M. Different metal doped TiO_2_ pillared montmorillonite has a wider ultraviolet absorption wavelength range than T/M, and the optical absorption ranges are extended from ultraviolet to visible light. The metal doped TiO_2_ pillared montmorillonite has a better photocatalytic ability. The addition of metal doped TiO_2_ pillared montmorillonite could also effectively increase the resistance to rutting and permanent deformation under high temperature conditions. In this study, metal doped modifiers were evaluated at the microscopic level by X-ray diffraction (XRD) and an Ultraviolet visible (UV-Vis) spectrophotometer [19]. The high temperature performance, UV aging resistance, and degradation of the automobile exhaust performance of metal doped modified bitumen were also studied. Metal doped modified asphalt has the potential to catalyze automobile exhaust and improve the aging resistance of asphalt. Metal doped, modified bitumen offers a certain contribution to the development of environmental protection and the long-life of asphalt pavement.

## 2. Materials and Methods

### 2.1. Materials

Sodium hydroxide (NaOH), nitric acid (HNO_3_), ethyl alcohol absolute (C_2_H_5_OH), tetra-n-butyl titanate (C_16_H_36_O_4_Ti), cupric nitrate (Cu(NO_3_)_2_), ferrous nitrate (Fe(NO_3_)_3_), cerium (III) nitrate hexahydrate (Ce(NO_3_)_3_), acetic acid (CH_3_COOH), and hexadecyl trimethyl ammonium bromide (CATB) were analytical grade and used without further purification. Na-montmorillonite clay (MMT, 90 meq/100 g) was collected from Xinyang, China. Original bitumen with a penetration grade of 70# (TZ70) was supplied by the China National Offshore Oil Corporation Taizhou Petroleum Asphalt Factory (Taizhou, China). The performance of the original bitumen is shown in Table 1.

### 2.2. Synthesis of TiO_2_ and Metal Doped TiO_2_ Pillared Montmorillonite

10 mL tetra-n-butyl titanate, 10 mL ethyl alcohol absolute, and 2 mL acetic acid were mixed for 20 min to form the transparent, yellowish Solution A. Preparation of Solution B begun with 24 mL HNO_3_ (1 mol/L) and 5 mL ethyl alcohol absolute. The light-yellow transparent sol of Ti was obtained by adding liquid A into liquid B with the aid of a separating funnel with intense magnetic stirring. The TiO_2_ pillared agent can be obtained by adjusting pH = 2 with a NaOH solution (1 mol/L) and continuous stirring.

2 g of MMT, which went through a 200 mesh sieve, was slowly added into 200 mL of deionized water under continuous stirring. The solution was subjected to ultrasonic vibration for 5 mins and then stirred continuously for 2 h with an electric agitator to form a suspension with a mass concentration of 1 wt %.

The prepared TiO_2_ pillared agent was continuously dripped into the suspension of MMT and stirred for 6 h at 65 °C. After being stirred completely, the solution was centrifuged to remove the separated upper liquid until the supernatant was near neutral, and the excess sol and other impurity ions were washed away. The precipitate was taken out and placed in a drying oven at 80 °C. The precursor of the TiO_2_ pillared montmorillonite (P-T/M) was obtained by being grinded through a 200 mesh sieve after the water was completely removed.

The pillared precursor was calcinated at 500 °C for 3 h with a heating rate of 2 °C/min. The TiO_2_ pillared montmorillonite (T/M) was obtained by grinding and passing the material through a 200 mesh sieve after being naturally cooled down to room temperature.

Metal doped TiO_2_ pillared montmorillonite was also prepared by the sol-gel method. The metals were doped to TiO_2_ with a molar mass ratio of [Ti^4+^/Metal ion] = 3/1. Corresponding Fe, Cu, and Ce metal salts were added to the above Solution A with the same procedure as the TiO_2_ pillared montmorillonite. The precursor of the metal doped TiO_2_ pillared montmorillonite was recorded as P-Cu-T/M, P-Fe-T/M, P-Ce-T/M, and the metal doped TiO_2_ pillared montmorillonite was recorded as Cu-T/M, Fe-T/M, Ce-T/M.

### 2.3. Preparation of Modified Bitumen

Different amounts (4 wt %, 5 wt %, and 6 wt %) of Cu-T/M, Fe-T/M, Ce-T/M, and T/M as bitumen modifiers were added into the 70# bitumen (TZ70) after being heated at 120 °C. Then, the bitumen was dispersed for 40 min under 140–155 °C with a rotation speed of 5000 rpm. Finally, the modified bitumen of the T/M, Cu-T/M, Fe-T/M, and Ce-T/M with different contents were prepared (named TZ70 + *X*% *Y*-T/M, *X* = 4, 5, 6, and *Y* for Cu, Fe, Ce). And 5 wt % of the T/M modified bitumen (TZ70 + 5% T/M) was selected for comparison.

### 2.4. Microscopic Tests of Samples

The phase identification of the MMT, T/M, and metal doped TiO_2_ pillared montmorillonite was investigated by X-ray diffraction (XRD) (Rigaku D/max 2550, Tokyo, Japan, Cu Kα radiation, 2θ = 0.5–80°. The UV-Vis spectra were determined by Shimadzu UV-2600 UV–Vis spectrophotometer (Kyoto, Japan) with a wavelength range of 300–1000 nm. The transmission electron microscopy (TEM) image of Ce-T/M was recorded by a JEOL JEM-2100 F electron microscope (Tokyo, Japan). The DM3000 fluorescence microscope (FM) was produced by Leica (Wetzlar, Germany), the excitation light was blue light, the eyepiece magnification was 10 times, the objective lens was adjustable in multiple gears, and the modified bitumen with 5 wt % Ce-T/M sample was tested.

### 2.5. High Temperature Rheological Tests

The high temperature rheological properties of the modified bitumen were tested with an MCR 301 intelligent dynamic shear rheometer (DSR) produced by Anton Paar (Graz, Austria). The test method is referred to AASHTO T 315-2012 [20,21]. Two parallel circular plates with a diameter of 25 mm and a spacing of 1 mm were used. The temperature ranged from 40 °C to 90 °C, with an increment of 2 °C/min. The oscillation frequency was 10 rad/s, and the strain values of the original sample and the ultraviolet aged sample were fixed at 12% and 10%, respectively. Three parallel samples were prepared, and the results were averaged. The complex shear modulus (G*) phase angle (δ) obtained from DSR can be used to calculate the resistance of the modified bitumen to rutting and permanent deformation under high temperature conditions. G*/sinδ is used as an indicator to reflect the permanent deformation of bitumen materials. From the perspective of anti-rutting, higher G* values and lower δ values are ideal.

### 2.6. Ultraviolet (UV) Aging Test

Prolonged UV radiation will cause the aging of bitumen, which will shorten the service life of the bitumen. The UV aging test aims to reveal the effects of Cu-T/M, Fe-T/M, Ce-T/M, and T/M on bitumen resistance to UV aging. 3.5 g of the modified bitumen or original bitumen was poured into a span with a diameter of 95 mm, resulting in a film thickness of about 1 mm. Then, the bitumen films were placed under a UV intensity of 3.18 W/m^2^ for 336 h to simulate three years of UV aging under an atmosphere. Finally, the residual bitumen sample after aging was analyzed by DSR. The aging index (AI) value is calculated according to the following formula:

(1) $$\mathrm{AI}=\frac{G_{aged}^{*}}{G_{fresh}^{*}}$$

where $G_{aged}^{*}$ is the complex shear modulus of bitumen after aging, and $G_{fresh}^{*}$ is the complex shear modulus of the fresh bitumen. The lower AI value reflects the better UV aging resistance of the bitumen.

### 2.7. Degradation Test of Automobile Exhaust

In the previous experiments, degradation tests of the automobile exhaust of modified bitumen were investigated by a self-designed UV environment simulation cabinet [5]. The environmental cabinet can effectively simulate the actual road environment and is equipped with an exhaust detection system (Gasboard-5030, Foshan Analyzer Co. Ltd., Foshan, China) to test the photocatalytic degradation of automobile exhaust. The instrument light source system adopts ultraviolet light emitting diode (UV-LED).

The average intensity of ultraviolet light in the experimental area was 0.52 W/m^2^. In order to speed up the experimental process, the UV intensity of this experiment was 10.4 W/m^2^, which was 20 times that of natural light. In this experiment, the dosage of the metal doped modifier was selected as 6 wt % and compared with the modified bitumen with 6 wt % T/M to determine the difference and promotion of the three metal doped modifiers. In this test, the irradiation time of all four samples was 90 min, the ambient temperature of the experimental cabinet was kept constant at 25 °C, and NO, HC was introduced to simulate the automobile exhaust environment. The composition and concentration of the automobile exhaust was the same as those of the previous experiments, according to the actual automobile exhaust [5]. A 60 g amount of modified bitumen was paved on a flat plate with dimensions of 20 mm × 30 mm, and the resultant thickness of the films was about 1 mm. Then, the plate of bitumen was put into the environmental cabinet, and the test was carried out at 25 °C for 90 min. The changes in concentration of NO, HC over this period were collected every 5 min [22,23].

In this paper, η is used as the degradation rate of gas, and the equation is:

(2) $$\eta =\frac{C_{0}-C_{1}}{C_{0}}\%$$

where *C*_0_ is the initial concentration of gas and *C*_1_ is the concentration of gas reaching the specified time.

The summary of samples and experiments are show in Table 2.

## 3. Results and Discussion

### 3.1. Microscopic Characterization of Samples

**Figure 1:**
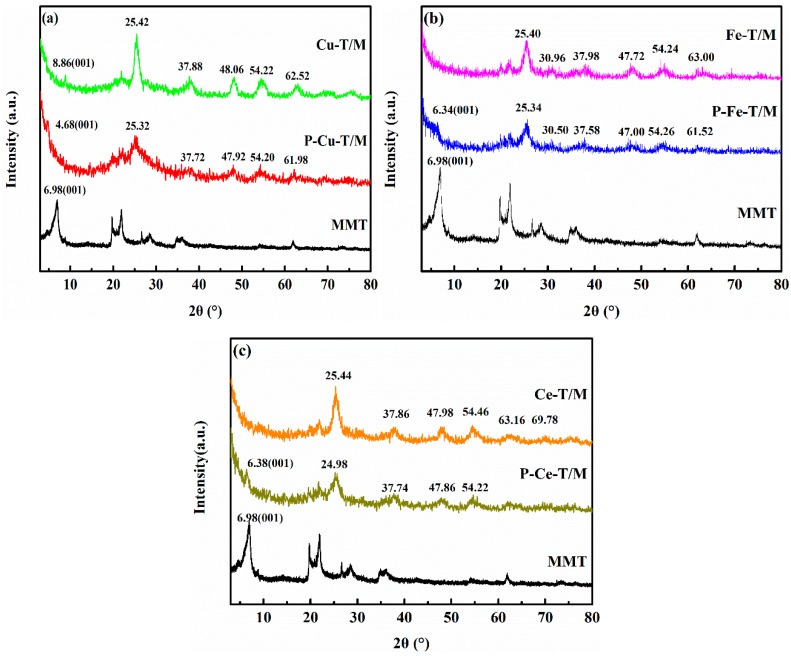
X-ray diffraction (XRD) patterns of MMT and (**a**) Cu-T/M, (**b**) Fe-T/M, (**c**) Ce-T/M with their precursor.

Figure 1 shows the XRD patterns of metal doped TiO_2_ pillared montmorillonite compared with those of MMT. As can be seen from Figure 1a, the (001) peak of MMT for P-Cu-T/M (observed at 2θ = 4.68°) is shifted forward compared with the (001) peak of MMT, which proves that the interlayer spacing of P-Cu-T/M is further expanded. Through the calculation of the Bragg equation (2dsinθ = nλ), the interlayer spacing of P-Cu-T/M is 1.89 nm and that of MMT is 1.27 nm. The (001) peak of Cu-T/M is observed at 2θ = 8.86°, which shifted backward when compared with that of MMT. The layer structure may collapse, and the interlayer spacing becomes 1.00 nm after calcination. The TiO_2_ characteristic peaks of the anatase phase are found at 2θ = 25.32°, 37.72°, and 54.20° for Cu-T/M, which shows that the TiO_2_ in Cu-T/M is mainly in the anatase phase. The characteristic peaks of Cu_2_O appeared at 2θ = 27.94°, 30.88°, 69.62°, and 75.1°, indicating that Cu-T/M was successfully synthesized.

Figure 1b shows the XRD patterns of P-Fe-T/M and Fe-T/M. The (001) peak of MMT for P-Fe-T/M is observed at 2θ = 6.34°, with interlayer spacing of 1.40 nm, which is 0.13 nm larger than that of original MMT. It indicates that the precursor of Fe-TiO_2_ could enter into the interlayer of MMT. But the characteristic (001) peak of MMT is not observed in Fe-T/M, which is due to the structure’s collapse by calcination. Fe-T/M showed characteristic peaks of anatase TiO_2_ at 2θ = 25.40°, 37.98°, 54.24°, rutile TiO_2_ at 2θ = 63.00°, and Fe_2_O_3_ at 2θ = 30.96°. The phase of TiO_2_ was not disturbed by Fe doping, as the radii of Ti^4+^ (0.068 nm) and Fe^2+^ (0.078 nm) ions are relatively close. Fe ions are easily doped into the crystal structure of TiO_2_ [24].

Figure 1c shows the XRD patterns of P-Ce-T/M and Ce-T/M. The (001) peak of MMT is observed at 2θ = 6.38° for P-Ce-T/M, which is shifted forward when compared with that of MMT. The interlayer spacing of P-Ce-T/M is 1.39 nm. P-Ce-T/M shows the characteristic peak of the anatase TiO_2_ at 2θ = 24.98°, 37.74°, 47.86°, and 54.22°. Ce-T/M showed characteristic peaks of the anatase TiO_2_ at 2θ = 25.44°, 37.86°, 47.98°, and 54.46°, with characteristic peaks of the rutile TiO_2_ appearing at 2θ = 63.16°, and the Ce_2_O_3_ characteristic peak appearing at 2θ = 69.78°. Ce-T/M has broad diffraction peaks with a higher peak height, which may be due to lattice distortion and a decrease in grain size of the TiO_2_ influenced by the doping of Ce. The unsaturated bonds and dangling bonds on the surface increase after the doping procedure; the surface effect is remarkable, and the photocatalyst surface has a strong chemical reactivity [25].

**Figure 2:**
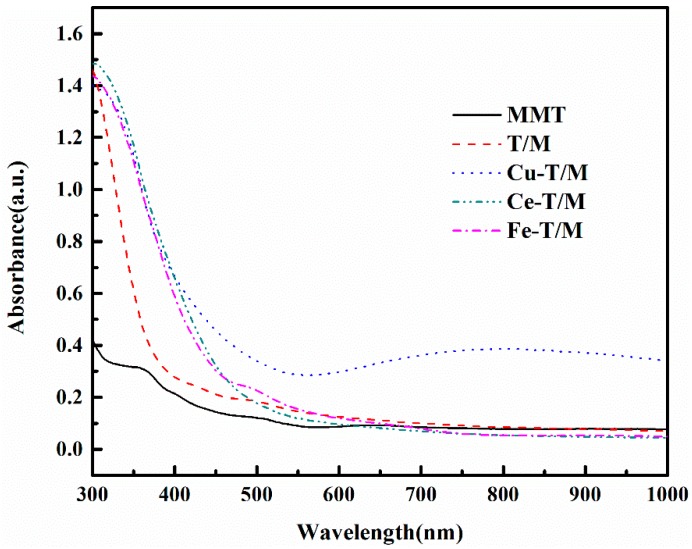
UV-Vis spectrum of metal doped TiO_2_ pillared montmorillonite.

The UV-vis spectrum of the metal doped TiO_2_ pillared montmorillonite are shown in Figure 2. All the absorbance curves of Cu-T/M, Fe-T/M and Ce-T/M move to the right with a noticeable red shift in the absorption edge after the TiO_2_ was doped with Cu, Fe, and Ce metals. Ion doping introduces specific metal ions into the lattice of the TiO_2_, which has a good effect on the formation and recombination of electron holes in the lattice. Some metal ions can replace Ti^4+^ in the lattice and promote the red shift of absorption wavelength. The specific surface area, adsorption performance and degradation ability of the lattice are enhanced [26,27,28]. Compared with the absorbance curve of T/M, the absorption range of ultraviolet light by Fe-T/M and Ce-T/M is extended to 300–500 nm, indicating the enhanced absorption capacity of UV light. The absorption wavelength range of ultraviolet light was clearly enlarged after being doped by Cu. Ce-T/M has a good absorption effect on ultraviolet light in a wavelength range of 300–600 nm and can still maintain better absorption capacity in the wavelength range of 600–1000 nm. Different metal doped TiO_2_ pillared montmorillonite has a wider ultraviolet absorption wavelength range than T/M, and the optical absorption ranges are extended from ultraviolet to visible light. The metal doped TiO_2_ pillared montmorillonite has a better photocatalytic ability, especially for Cu-T/M [29,30].

**Figure 3:**
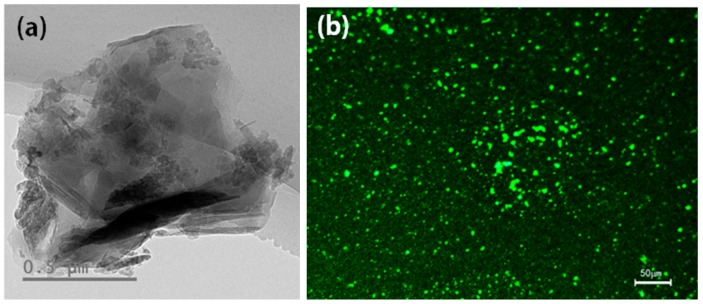
(**a**) TEM image of Ce-T/M powder and (**b**) fluorescence microscope (FM) image of 5 wt % Ce-T/M modified bitumen.

The morphology of Ce-T/M was analyzed by TEM image (Figure 3a). The typical parallel structure of montmorillonite with a 1 μm in diameter was observed. The dark particles in the Ce-MMT is concluded to be the Ce-TiO_2_ on the surface or in the layer of MMT. FM was carried out on the 5 wt % Ce-T/M modified bitumen to observe the distribution of the Ce-TiO_2_ nano powder in the bitumen (Figure 3b). The bitumen of the image is displayed as black with a continuous phase, and the Ce-TiO_2_ nano powder is shown as green bright spots by adjusting the brightness.

### 3.2. High Temperature Rheological Properties of Modified Bitumen

**Figure 4:**
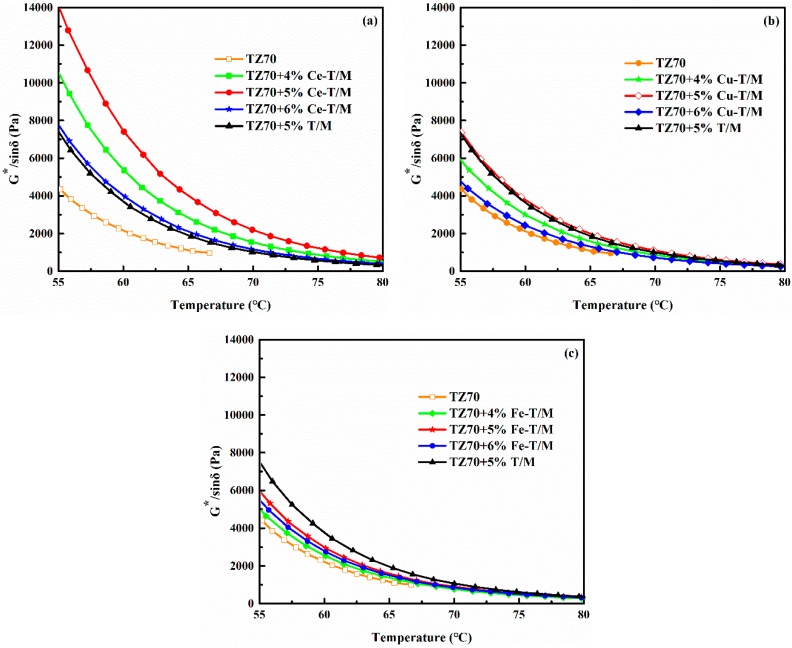
Variation of the G*/sinδ value of (**a**) Ce-T/M, (**b**) Cu-T/M, (**c**) Fe-T/M modified bitumen at different temperatures.

The high temperature rheological properties of the metal doped modified bitumen were tested by DSR. The content of the three modifiers in the bitumen were 4 wt %, 5 wt %, and 6 wt %; the modified bitumen with a 5 wt % T/M was selected as a contrast (Figure 4). The G*/sinδ of the bitumen is obviously improved after being modified with Cu-T/M, Fe-T/M, and Ce-T/M. The exfoliation structure was formed after adding montmorillonite to the bitumen, and the modifier was uniformly dispersed in the bitumen. Large numbers of bitumen particles entered the montmorillonite layer, and the compatibility between the modifier and asphalt increased, resulting in improved high temperature rheological properties for the asphalt [31]. It can be found that the G*/sinδ of the 5 wt % Cu-T/M and Ce-T/M modified bitumen is bigger than that of the 5 wt % T/M modified bitumen. However, the G*/sinδ of the Fe-T/M modified bitumen is less than that of that of the 5 wt % T/M modified bitumen. It was proven that the high temperature rheological properties of the T/M modified bitumen can be further improved by being doped with two metal ions of Ce and Cu, but the effect of Fe ion is not obvious. A lateral comparison of the curves in Figure 4a–c show that the G*/sinδ of the Ce-T/M modified bitumen at different temperatures is higher than that of the Cu-T/M and Fe-T/M modified bitumen with the same content, which proves that the high temperature performance of the Ce-T/M modified bitumen is obviously improved. The rutting factor G*/sinδ of the modified bitumen increased significantly with an increase of nano-metal oxides, which means that the viscous properties of the bitumen are weakened, and the high temperature resistance to permanent deformation is significantly enhanced [32].

**Figure 5:**
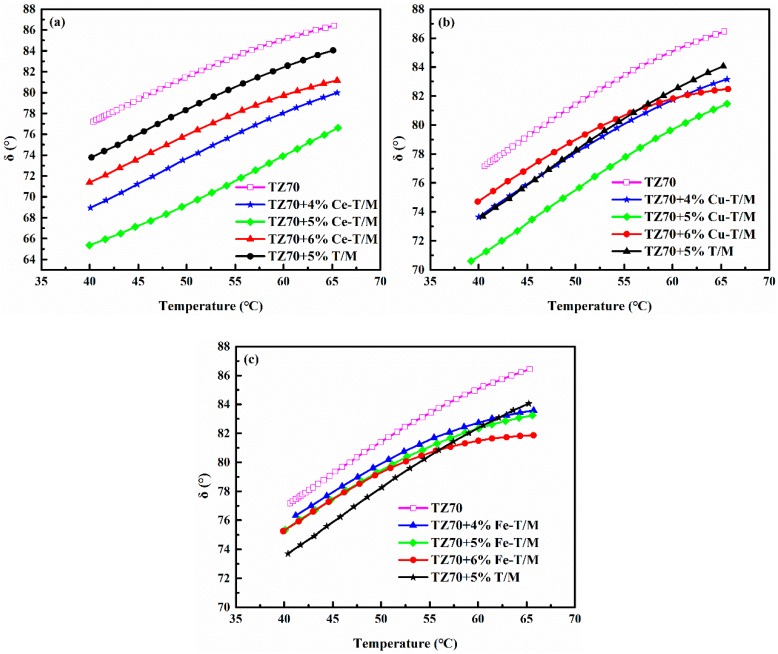
Variation of δ value of (**a**) Ce-T/M, (**b**) Cu-T/M, and (**c**) Fe-T/M modified bitumen at different temperatures.

The δ of the three modifiers is the smallest at different temperatures when the content of the three modifiers is 5 wt % (Figure 5). The δ values under the different temperatures of the Cu-T/M and Ce-T/M modified bitumen at 5 wt % dosage are lower than those of the 5 wt % T/M modified bitumen. This result shows that the viscoelastic properties of the T/M modified bitumen are improved by being doped with Ce and Cu, indicating strong resistance to high temperature deformation. Table 3 shows the critical high temperature of different modified bitumen samples. Compared with the 5 wt % T/M, the c critical high temperature of the 5 wt % Ce-T/M modified bitumen increased by 7.79 °C, and the 5 wt % Cu-T/M modified bitumen increased by 2.76 °C. The improvement of the high temperature rheological properties of the Cu-T/M and Ce-T/M modified bitumen is related to the Cu-TiO_2_ and Ce-TiO_2_ formed by doping. An exfoliated structure of Cu-T/M and Ce-T/M is also formed in the bitumen, which increases the rutting resistance of the bitumen at high temperatures and slows down the flow performance at high temperatures [33].

### 3.3. UV Aging of Modified Bitumen

**Figure 6:**
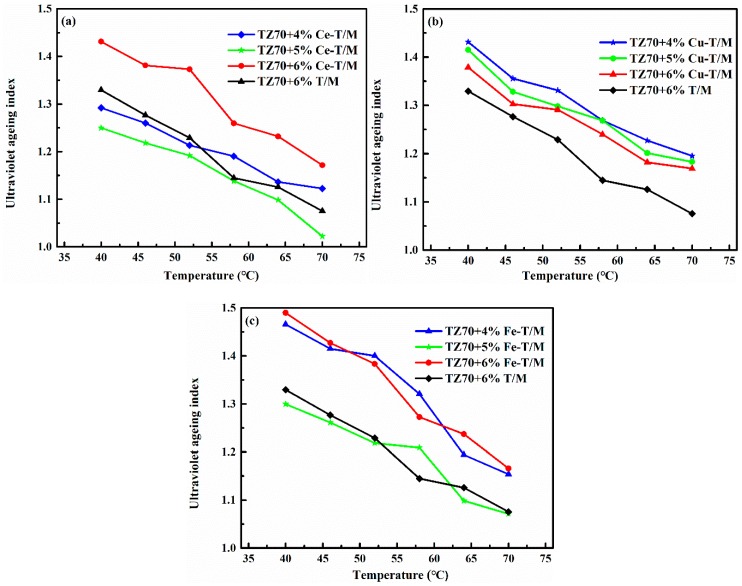
The AI value of (**a**) Ce-T/M, (**b**) Cu-T/M, and (**c**) Fe-T/M modified bitumen after UV aging.

The optimum dosage and effects of different modifiers on UV aging resistance were investigated by UV aging tests. After UV aging, the residual bitumen samples were analyzed by DSR. Then, the G* values of the Cu-T/M, Fe-T/M, and Ce-T/M modified bitumen at different temperatures were obtained, and the AI values of the Cu-T/M, Fe-T/M, and Ce-T/M modified bitumen were calculated [31,34]. The optimum dosage of the Cu-T/M, Fe-T/, and Ce-T/M is 5 wt % in the temperature range of 40–70 °C. A horizontal comparison of the AI values of the 5 wt % Cu-T/M, Fe-T/M, and Ce-T/M modified bitumen is shown in Figure 6. The AI value of the Ce-T/M modified bitumen at different temperatures is lower than the other two, which is closer to 1. This analysis indicated that the degree of UV aging is lower after being modified with 5 wt % Ce-T/M, and the effect of improving the UV aging resistance of bitumen is the best. According to the analysis shown in Figure 2 and Figure 4, Ce-T/M has the best UV shielding ability and high temperature rheological properties, so the UV aging resistance ability of the Ce-T/M modified asphalt is the most obvious. Compared with T/M, the UV absorption capacities of Fe-T/M and Cu-T/M were enhanced, but the high temperature rheological properties of the Fe-T/M and Cu-T/M modified asphalt were degraded, so the UV aging resistance of the Fe-T/M and Cu-T/M modified asphalts did not improve [25,33].

### 3.4. Photocatalytic Degradation of Tail Gas Test

**Figure 7:**
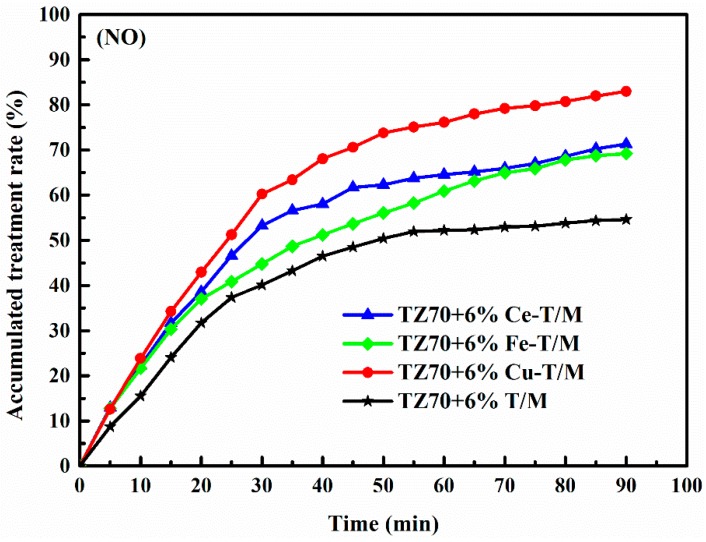
Degradation curve of NO by four modified bitumen.

In the experiment of degradation exhaust gases, the blank control groups were set up firstly. Because the original bitumen did not possess the photocatalytic capability to calculate the bare degradation rate, the degradation rates of the exhaust gases catalyzed by the original bitumen were first measured at the same experimental time and temperature. The degradation rates of the exhaust gases catalyzed by the metal doped TiO_2_ pillared montmorillonite minus the blank control groups values were measured to obtain the bare degradation rates. Figure 7 shows the NO degradation curve after subtracting the blank group. The maximum degradation rate (η) of the 6 wt % Cu-T/M, Ce-T/M, and Fe-T/M modified bitumen reached 82.99%, 71.30%, and 69.20% after 90 min, respectively, while that of the T/M modified bitumen at 90 min was 54.63% for the NO. Cu-T/M modified bitumen had the strongest degradation ability among NO gases. Compared to the T/M modified bitumen, the Fe-T/M modified bitumen showed no obviously increased degradation for the NO gas. Doping Cu, Ce can efficiently enhance the degradation for NO gases of TiO_2_. The η growth of the four kinds of modified bitumen is faster at 0–30 min, and becomes slower at 30–90 min. The degradation ability of the T/M modified bitumen was saturated after 55 min, but the Cu-T/M and Ce-T/M modified bitumen maintained a high growth rate in the 30–90 min period. The degradation rate of the Cu-T/M modified bitumen was faster, indicating the stronger catalytic degradation of the NO gas [25,35].

**Figure 8:**
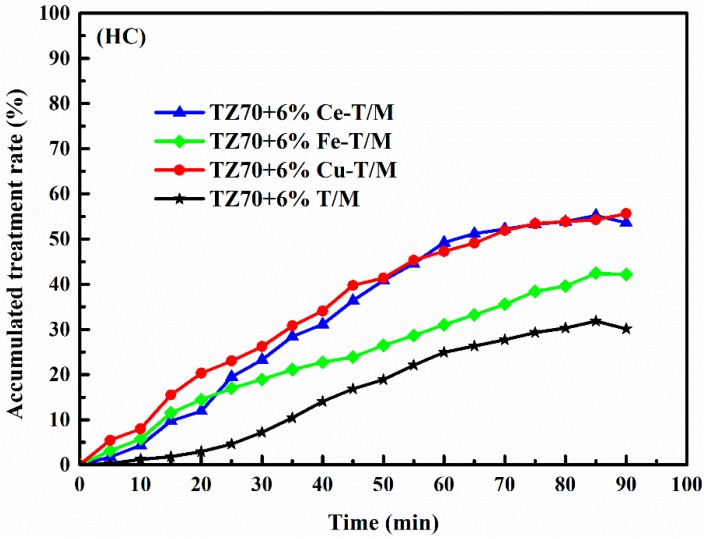
Degradation curve of HC by four modified bitumen.

Figure 8 shows the HC degradation curve after subtracting the blank group. The degradation rate of the HC gas gradually increases as the reaction time increases. This is mainly due to the oxidation-reduction reaction of the hydroxyl radicals (–OH) and superoxide radicals (–O^2−^) on the surface of Cu-T/M, Fe-T/M, Ce-T/M, and T/M under ultraviolet irradiation. After 90 minutes, the η of Cu-T/M, Ce-T/M, Fe-T/M, and T/M modified bitumen reached 55.59%, 53.60%, 42.19%, and 30.14%, respectively. The final degradation rate of the Cu-T/M, Fe-T/M, and Ce-T/M modified bitumen was higher than that of the T/M modified bitumen. The degradation rate is ranked as Cu-T/M > Ce-T/M > Fe-T/M > T/M, indicating that after the TiO_2_ is doped with Cu, Ce, and Fe, the redox ability of the crystal surface under UV irradiation is enhanced [36].

Compared to the T/M modified bitumen, the Cu-T/M, Fe-T/M, and Ce-T/M modified bitumen exhibited obvious improvement in UV aging resistance and photocatalytic activity to HC and NO gases after doping TiO_2_ with Ce, Cu, and Fe. This was mainly due to the obvious red shift of the optical absorption edge of the three kinds of modified bitumen, which made the optical absorption range extend from an ultraviolet region to a visible region. The red shift of Cu-T/M, Fe-T/M, and Ce-T/M is attributed to the charge transfer transition between the three metal ion electrons of Ce, Cu, and Fe, and the conduction band or valence band of TiO_2_, resulting in a decrease in the band gap energy of TiO_2_. After doping Ce, Cu, and Fe into T/M, the unit cell expanded, and the lattice became distorted. The positive and negative charges moved to the center, the degenerate orbit decreased, and the number of excited electrons increased, which promoted the capture of photogenerated electrons, and then the electron-hole pairs increased. All these phenomena resulted in the improved photocatalytic capacity of Cu-T/M, Fe-T/M, and Ce-T/M modified bitumen [37]. Cu-T/M displayed superior photocatalytic capacity for NO and HC, which may be due to the similar atomic radius for Cu^2+^ (0.073 nm) and Ti^4+^ (0.068 nm). Compared with Ce^4+^ (0.102 nm) and Fe^2+^ (0.078 nm), Cu^2+^ can easily enter the lattice of TiO_2_, which induces more electron-hole pairs and better catalytic performance. Metal oxides with high activity, such as Cu, can achieve similar catalytic effects to rare metals at normal temperatures. Moreover, Cu could be widely applied because of its lower cost, and because it has practical road engineering applications, which provide a new research direction for the metal doped catalytic degradation of automobile exhaust [23,38].

## 4. Conclusions

This work mainly studies the effects of metal doped TiO_2_ pillared montmorillonite on bitumen, including a physical characterization of the modifiers, high temperature properties, and UV aging resistance of modified bitumen. Furthermore, the photocatalytic degradation of tail gas properties by modified bitumen was investigated. Several major conclusions are as follows:

(1) The interlayer spacing of Cu-T/M, Fe-T/M, and Ce-T/M was further expanded compared to that of MMT, indicating that Cu-T/M, Fe-T/M, and Ce-T/M were successfully synthesized.
(2) The optical absorption edges of Cu-T/M, Fe-T/M, and Ce-T/M were red shift, and the UV absorption capacity was enhanced compared to that of T/M. Further, the absorption range of the metal doped modified bitumen was extended from UV to visible light, which improved the UV aging resistance and photocatalytic properties of the modified bitumen. The red shift of absorption edge of Cu-T/M is the most obvious, and the absorption range of ultraviolet is the most extensive.
(3) After the three different dosage modifiers for Cu-T/M, Fe-T/M, and Ce-T/M were added to the bitumen, the G*/sinδ of the modified bitumen was significantly improved at different temperatures, while the δ decreased. The high temperature rheological properties of the three kinds of modified bitumen were optimal with the dosage of 5 wt %, and Ce-T/M features the best high temperature rheological properties among the three kinds of modified bitumen.
(4) 5 wt % Ce-T/M modified bitumen had the lowest AI value at different temperatures in the temperature range of 40–70 °C, indicating the best UV aging resistance for the bitumen.
(5) The degradation rate of NO and HC gas is obviously improved by doping with Cu, Ce, and Fe. The degradation order is: Cu-T/M > Ce-T/M > Fe-T/M > T/M. Cu-T/M exhibits the best excellent photocatalytic performance for HC and NO gases.
(6) Only the degradation rate of modified bitumen at 25 °C was studied in this paper, and the effect of different environments on the degradation rate of modified bitumen was not considered. The next step will aim at studying the effects of different temperatures and humidity on the degradation rate of modified bitumen.

## Figures and Tables

**Table 1:** Physical properties of 70# original bitumen.

Properties	Limitation	Values
Penetration (25 °C, 100 g, 5 s), 1/10 mm	60~80	68.2
Penetration index	−1.5~+1.0	−1.06
Softening point, °C	≥46	47.3
Ductility (15 °C, 5 cm/min), cm	≥100	>148
Dynamic viscosity (60 °C), Pa·s	≥180	195
Density (15 °C), g/cm^3^	—	1.026

**Table 2:** The summary of samples and experiments in the paper.

Sample Types	X-ray Diffraction (Powder)	UV-Vis Spectra (Powder)	High Temperature Rheological (Bitumen)	UV Aging (Bitumen)	Automobile Exhaust Degradation (Bitumen)
Original bitumen			√		√
MMT	√	√			
T/M		√	√	√	√
Ce-T/M	√	√	√	√	√
Cu-T/M	√	√	√	√	√
Fe-T/M	√	√	√	√	√

**Table 3:** Critical high temperature for different bitumen samples.

Bitumen Types	Critical High Temperature	Temperature Change
Original bitumen	65.37 °C	—
5 wt % T/M	70.29 °C	+4.92 °C
4 wt % Ce-T/M	73.81 °C	+8.44 °C
5 wt % Ce-T/M	78.08 °C	+12.71 °C
6 wt % Ce-T/M	71.39 °C	+6.02 °C
4 wt % Cu-T/M	69.06 °C	+3.69 °C
5 wt % Cu-T/M	73.05 °C	+7.68 °C
6 wt % Cu-T/M	67.54 °C	+2.17 °C
4 wt % Fe-T/M	66.02 °C	+0.65 °C
5 wt % Fe-T/M	67.25 °C	+1.88 °C
6 wt % Fe-T/M	66.80 °C	+1.43 °C
